# Distribution and Genetic Diversity of *Borrelia* from Lyme Borreliosis and Relapsing Fever Groups in Ixodidae Ticks in the Russian Far East

**DOI:** 10.3390/pathogens15070775

**Published:** 2026-07-22

**Authors:** Yuliya Sabitova, Vera Rar, Valeria Fedorets, Yana Igolkina, Tamara Epikhina, Aleksey Nikitin, Yulia Verzhutskaya, Valentina Kolesnikova, Natalia Gordeiko, Natalya Pukhovskaya, Nina Tikunova

**Affiliations:** 1 Knorre Institute of Chemical Biology and Fundamental Medicine, Siberian Branch of the Russian Academy of Sciences, 630090 Novosibirsk, Russia; yvsabitova@mail.ru (Y.S.); v.fedorets@alumni.nsu.ru (V.F.); igolkina@inbox.ru (Y.I.); tikunova@1bio.ru (N.T.); 2 Irkutsk Antiplague Research Institute of Rospotrebnadzor, 664047 Irkutsk, Russia; nikitin_irk@mail.ru (A.N.); linika@mail.ru (Y.V.); valyusha.kolesnikova.92@mail.ru (V.K.); 3 Primorsk Antiplague Station of Rospotrebnadzor, 692512 Ussuriysk, Russia; gordeyko_ns@mail.ru; 4 Khabarovsk Antiplague Station of Rospotrebnadzor, 680031 Khabarovsk, Russia

**Keywords:** *Borrelia afzelii*, *Borrelia bavariensis*, *Borrelia garinii*, *Borrelia miyamotoi*, relapsing fever group, *Ixodes* spp., *Haemaphysalis* spp., Russian Far East, phylogenetic analysis

## Abstract

Ixodidae ticks are vectors of pathogenic spirochetes from the *Borrelia burgdorferi* sensu lato (s.l.) complex and the relapsing fever (RF) group. The Russian Far East is endemic for borrelioses, yet data on the distribution and genetic diversity of *Borrelia* spp. in this region remain scarce. We examined 3107 Ixodidae ticks (*Ixodes* spp., *Haemaphysalis* spp., and *Dermacentor silvarum*), collected from two mainland provinces and five islands of the Russian Far East in 2005–2023. A total of 86 *Borrelia afzelii*, 297 *Borrelia bavariensis*, 81 *Borrelia garinii*, 44 *Borrelia miyamotoi*, and 33 unclassified RF borreliae were identified. *Borrelia burgdorferi* s.l. and *B. miyamotoi* were strongly associated with *Ixodes* spp., whereas unclassified RF borreliae were associated with *Haemaphysalis* spp. Among *B. burgdorferi* s.l. isolates, *B. afzelii* and *B. bavariensis* were found significantly more frequently in *I. persulcatus* compared to *I. pavlovskyi*, whereas *B. garinii* was significantly more common in *I. pavlovskyi*. Genetic characterization of *B. burgdorferi* s.l. revealed high haplotypic diversity, particularly within *B. bavariensis* and *B. garinii*. Among RF borreliae, all *B. miyamotoi* isolates were highly conserved and belonged to the Asian type. In contrast, unclassified RF borreliae were genetically variable and clustered with RF borreliae previously found in *Haemaphysalis* and *Rhipicephalus* ticks. The obtained results expand the understanding of the distribution and genetic diversity of *Borrelia* spp. in the Russian Far East.

## 1. Introduction

Gram–negative free-living spirochetes of the genus *Borrelia* (order Spirochaetales, family Borreliaceae) are genetically heterogeneous microorganisms that differ in their biological and pathogenic properties. *Borrelia* species have been divided into three main groups: the Lyme borreliosis (LB) group (*Borrelia burgdorferi* sensu lato complex), the relapsing fever (RF) group, and the more recently recognized Echidna-Reptile (REP) group [1,2,3,4,5,6]. Several candidate species (e.g., “*Candidatus* Borrelia africana” and “*Candidatus* Borrelia ivorensis” associated with *Amblyomma* spp.) remain unassigned to any of these groups [1,3].

The LB group includes at least 23 validated and three proposed species. Six of these are confirmed agents of LB, and three species are potentially associated with human infection [2]. The four most epidemiologically important species of the LB group include *B. burgdorferi* s.s., whose distribution is largely limited to North America, as well as *B. afzelii*, *B. bavariensis*, and *B. garinii*, which are widespread across Eurasia. All *Borrelia* of this group are primarily associated with *Ixodes* spp. [7]. LB spirochetes are effectively transmitted transstadially but not transovarially. Reservoir hosts differ for various species and include mainly small mammals and birds [8].

The RF group is more diverse and includes 22 validated and several proposed species [1,3]. Depending on the vector, RF borreliae are subdivided into soft-tick-borne relapsing fever (STBRF), hard-tick-borne relapsing fever (HTBRF), and louse-borne relapsing fever (LBRF) groups [2]. In contrast to *B. burgdorferi* s.l., spirochetes from the RF group are effectively transmitted transovarially by ticks but not by lice [3,9,10].

The LBRF group is represented by a single highly pathogenic species—*Borrelia recurrentis*. Based on geographic distribution, STBRF borreliae are classified into “Old World” and “New World” strains. In the Old World, the disease is primarily caused by *B. hispanica*, *B. persica*, *B. duttonii*, and *B. crocidurae*, whereas in the New World, *B. hermsii*, *B. parkeri*, and *B. turicatae* are the predominant pathogens [3]. The HTBRF group includes three recognized species: *Borrelia theileri*, *Borrelia lonestari*, and *Borrelia miyamotoi* [11,12]. *Borrelia miyamotoi* is the only human pathogen from this group. It is mainly transmitted by *Ixodes* spp. and has the same distribution areas and vector range as LB spirochetes [13]. Genetically, *B. miyamotoi* isolates fall into four clusters, differing by geography and vectors—American type (*Ixodes scapularis*/*Ixodes pacificus*), European type (*Ixodes ricinus*), Siberian or Asian type (*Ixodes persulcatus*/*Ixodes pavlovskyi*), and *Ixodes ovatus* type (*I. ovatus*) [12,14,15]. In addition to recognized species, isolates genetically similar to *B. theileri* were found in *Haemaphysalis* spp. in Japan and China [10,16] and *Rhipicephalus* spp. in Iran and Portugal [17,18]. Moreover, *Borrelia* sp. HM strain has been cultured from *Haemaphysalis megaspinosa* in Japan and characterized by complete genome sequencing [10,19].

The REP group includes *Borrelia turcica* from *Hyalomma aegyptium* on turtles in Turkey [20], *Borrelia tachyglossi* from *Bothriocroton concolor* on echidnas in Australia [5,21], and “*Candidatus* Borrelia mahuryensis” associated with passerine birds [4], along with several unclassified isolates [1,2]. These species are transmitted by hard ticks, and their pathogenic potential is unknown.

The territory of southern Asian Russia and neighboring East Asian countries (Mongolia, China, Korea, and Japan) is endemic for LB [22]. Three species of *B. burgdorferi* s.l. dominate in this territory: *B. afzelii*, *B. bavariensis*, and *B. garinii* [23,24,25,26,27,28,29]. However, reports on the prevalence and species composition of *B. burgdorferi* s.l. in East Asia are frequently inconsistent, reflecting variation in study year, sampling location, and detection methods [30,31]. Several studies have shown that pathogenic LB agents in Northeastern Asia are mainly harbored by *I. persulcatus*, *I. granulatus*, and *I. pavlovskyi* [23,24,25,28,29,31,32,33]; in contrast, non-pathogenic *Borrelia japonica* has been detected in *Ixodes ovatus* [29]. In addition to LB spirochetes, *B. miyamotoi* from the RF group has been identified in *I. persulcatus* and *I. pavlovskyi* across various locations in Asian Russia and East Asian countries [15,23,34].

The Russian Far East is characterized by high species diversity of Ixodidae ticks belonging to the genera *Ixodes*, *Haemaphysalis*, and *Dermacentor* [35,36,37,38]. The most common tick species are *I. persulcatus*, *I. pavlovskyi*, *H. concinna*, *H. japonica*, *H. longicornis*, and *D. silvarum*. Notably, unlike other regions of the Asian part of Russia, the occurrence and distribution of *B. burgdorferi* s.l. species and borreliae from the RF group in the Russian Far East remain largely unexplored. To address this gap, we investigated the prevalence, species diversity, and genetic variability of *Borrelia* from both the LB and RF groups in Ixodidae ticks collected from different sites of the Russian Far East. These sites included both mainland locations and islands, which differed markedly in their tick species composition. The sampling period spanned nearly two decades, allowing us to assess the historical distribution patterns of *Borrelia* spp. in the region.

## 2. Materials and Methods

### 2.1. Sampling Sites

**Figure 1:**
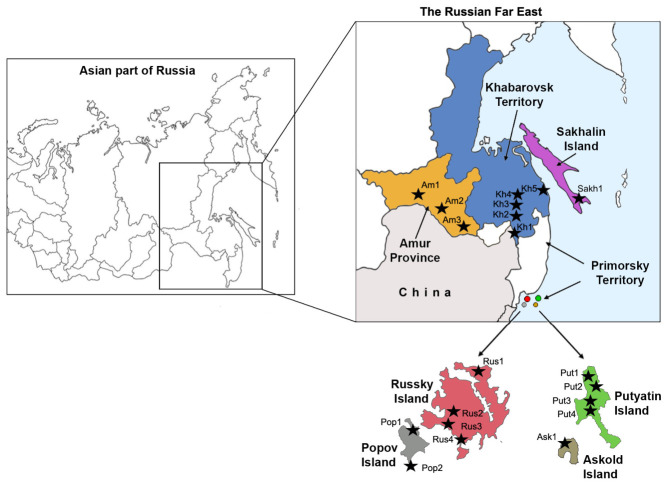
The map of the Russian Far East showing the location of sampling sites. Asterisks indicate the sites of tick collections.

Questing ixodid ticks were collected from vegetation using a white cotton cloth (60 × 100 cm) in 20 sampling sites located in forest biotopes in the Khabarovsk Territory, Amur province, as well as on Russky, Putyatin, Askold, Popov, and Sakhalin islands (Figure 1, Table S1). Collections were conducted during the peak of seasonal activity in May from 2005 to 2023. Russky, Putyatin, Askold, and Popov islands are located in Peter the Great Bay of the Sea of Japan (Primorsky Territory), whereas Sakhalin, the largest of the Russian islands in the Far East, is bordered by the Sea of Okhotsk and the Sea of Japan.

### 2.2. Tick Species Determination

To minimize the risk of cross-contamination, all steps involving DNA extraction, PCR assay, and gel electrophoresis were performed in separate, dedicated laboratory spaces. Tick genus and developmental stage were determined morphologically [39,40] using a stereo microscope MC-2 (Biomed, Saint Petersburg, Russia). Following morphological examination, ticks were washed sequentially in bidistilled water, 70% ethanol, and again in bidistilled water and then homogenized using the MagNA Lyser system (Roche Diagnostics, Basel, Switzerland). Total DNA was obtained from tick homogenates using the commercial Proba NK extraction kit (DNA-Technology, Moscow, Russia) in accordance with the manufacturer’s guidelines. For *Ixodes* spp. females collected in *I. persulcatus*/*I. pavlovskyi* sympatric areas, DNA was extracted separately from capitula and bodies. DNA from capitula was used to accurately identify tick species to exclude misidentification of female *Ixodes* spp. fertilized by a male of another species, whereas DNA from bodies was used for *Borrelia* spp. detection.

The species of *Ixodes* spp. and *Haemaphysalis* spp. were determined using species-specific PCR targeting the mitochondrial cytochrome c oxidase subunit 1 (*cox*1) gene fragments of *I. persulcatus*, *I. pavlovskyi*, *H. concinna*, *H. japonica*, and *H. longicornis* (Table 1), as previously described [23,41]. All *Ixodes* spp. ticks collected in *I. persulcatus*/*I. pavlovskyi* sympatric areas were additionally examined for the presence of *I. persulcatus*/*I. pavlovskyi* interspecific hybrids (hereinafter referred to as hybrids) by amplification and sequencing of the nuclear internal transcribed spacer 2 (ITS2) fragments (Table 1), as previously described [42]. Ticks displaying heterozygosity in ITS2 fragments at positions that are diagnostic for *I. persulcatus* and *I. pavlovskyi* were classified as ticks carrying hybrid ITS2 fragments. *Ixodes* spp. ticks with hybrid ITS2 fragments, as well as those showing discordance between mitochondrial and nuclear loci, were designated as first-generation (F1) and second-generation (F2) hybrids, respectively.

### 2.3. Detection and Genetic Study of Borrelia burgdorferi Sensu Lato

*Borrelia burgdorferi* s.l. was detected in individual ticks by nested PCR targeting the 5S-23S rRNA intergenic spacer (IGS). Positive samples were further processed by nested PCR to obtain the *clp*A and/or *p83/100* gene fragments using the primers listed in Table 1.

PCR was carried out in a final volume of 20 µL containing 1× PCR buffer, 200 µM of each dNTP, 2 U of *Taq* DNA polymerase (Biolabmix, Novosibirsk, Russia), 0.5 µM of each primer, and 2 µL of template DNA (or 2 µL of primary PCR product for nested reactions). Amplification was performed under the following conditions: initial denaturation at 94 °C for 3 min; 35 cycles of 94 °C for 30 s, annealing at the temperatures indicated in Table 1 for 30 s, and 72 °C for 1 min; and a final extension at 72 °C for 5 min. Sterile double-distilled water was included as a negative control in each run. Positive controls consisted of DNA from *B. afzelii* (strain Tom1303) and *B. garinii* (strain Tom3005), which had been isolated from *Ixodes* spp. in 2003 and 2005, respectively, and genotyped by multilocus sequence typing (MLST) [43].

To identify mixed infections and differentiate *B. afzelii* from *B. bavariensis* and *B. garinii*, the sizes of obtained PCR fragments of the *p83/100* gene were compared; *B. afzelii* yielded 336 bp fragments, whereas *B. bavariensis* and *B. garinii* produced 426 bp fragments [23]. The presence of both bands indicates a mixed infection. Species identification and genetic analysis of positive samples were performed by bidirectional sequencing of the *clp*A and/or *p83/100* gene fragments.

### 2.4. Detection and Genetic Study of Borrelia from the RF Group

*Borrelia* from the RF group were identified in individual ticks by nested PCR targeting the *glp*Q gene, using the primers indicated in Table 1. DNA of *B. miyamotoi*, previously identified in *Ixodes* spp. and genotyped by sequencing the *glp*Q gene [23], was used as a positive control, and sterile double-distilled water was used as a negative control. Species identification of RF borreliae was based on sequencing of the *glp*Q gene fragments. For further sequencing, the positive specimens were additionally amplified for the *fla*B and 16S rRNA gene fragments (Table 1).

**Table 1:** Primers used for detection of Ixodidae ticks and *Borrelia* spp.

Locus	Organism	Reaction	Primer Name	Primer Sequences 5′-3′	Size (bp)	T * (°C)	References
*cox*1	*I. persulcatus*	conventional	Ixodes-F	acctgatatagctttccctcg	690	55	[23]
			Ipers-R	ttgattcctgttggaacagc			
	*I. pavlovskyi*	conventional	Ixodes-F	acctgatatagctttccctcg	689	55	[23]
			Ipav-R	taatccccgtggggacg			
	*H. japonica*	conventional	Hj-1	ggyacttgagctggaatattaggctt	629	57	[41]
			Hj-2	tggtataaaattggatccccgcc			
	*H. concinna*	conventional	Hc-1	gracttgagcaggaatactaggrtt	629	57	[41]
			Hc-2	tgatataaaattgggtcacctcc			
	*H. longicornis*	conventional	Hlong-F	ttgagccggaatgctaggtctaag	853	60	This study
			Hlong-R	ggctgaagtaaagtaggcccg			
ITS2	Ixodidae	conventional	F-ITS2	cacactgagcacttactctttg	632–636	57	[23]
			R-ITS2	actggatggctccagtattc			
IGS	*B. burgdorferi* s.l.	primary	NC1	cctgttatcattccgaacacag	350–384	50	[23]
			NC2	tactccattcggtaatcttggg			
		nested	NC3	tactgcgagttcgcgggag	237–271	50	
			NC4	cctaggcattcaccatagac			
*clpA*	*B. burgdorferi* s.l.	primary	clpA1	aaagatagatttcttccagac	982	50	[23]
			clpA2	gaatttcatctattaaaagctttc			
		nested	clpA3	gacaaagcttttgatattttag	850	50	
			clpA4	caaaaaaaacatcaaattttctatctc			
*p83/100*	*B. burgdorferi* s.l.	primary	F7	ttcaaagggatactgttagagag	438–528	50	[23]
			F10	aagaaggcttatctaatggtgatg			
		nested	F5	acctggtgatgtaagttctcc	336–426	54	
			F12	ctaacctcattgttgttagactt			
*glpQ*	RF *Borrelia*	primary	Q1	caccattgatcatagctcacag	633	50	[23]
			Q4	ctgttggtgcttcattccagtc			
		nested	Q3	gctagtgggtatcttccagaac	424	54	
			Q2	cttgttgtttatgccagaagggt			
*flaB*	RF *Borrelia*	primary	fla1	ctgatgatgctgctggtatgggtg	844–862	55	This study
			fla4	tgaggcacttgatttgcttgtgc			
		nested	fla3	caggctcaatataaccagatgca	455–473	50	
			fla2	tcaatagcataatctgtgctagc			
16S rRNA	*Borrelia* spp.	primary	S1	gctggcagtgcgtcttaagcatgc	1367	50	[44]
			S2	gtgacgggcggtgtgtacaaggccc			
		nested	S7	gtggcgaacgggtgagtaacgcg	1292	50	This study
			S6	gatacggtgaatacgttctcggg			

* Annealing temperature.

### 2.5. Sequencing and Phylogenetic Analysis

The obtained PCR products were separated on 0.6% SeaKem^®^ GTG-agarose gels (Lonza, Haifa, Israel), following excision of the target bands. Sanger sequencing was performed in both directions with the BigDye Terminator v.3.1 Cycle Sequencing Kit (Applied Biosystems, Carlsbad, CA, USA), and the sequencing products were resolved on an ABI 3500 Genetic Analyzer (Applied Biosystems Inc.).

Sequences were compared with reference data in the NCBI database using BLASTN (https://blast.ncbi.nlm.nih.gov), accessed on 20 April 2026, and multiple sequence alignments were generated with MEGA 7.0. (www.megasoftware.net). The *clpA* alleles of *B. burgdorferi* s.l. were further compared with those deposited in the PubMLST database (https://pubmlst.org/organisms/borrelia-spp), accessed on 20 April 2026. Phylogenetic relationships were reconstructed using the maximum-likelihood (ML) method in MEGA 7.0, with the best-fit substitution model selected using the Bayesian Information Criterion (BIC) [45].

### 2.6. Statistical Analysis

Differences in *Borrelia* prevalence among tick species were assessed using the Fisher’s exact test (two-tailed) (http://www.socscistatistics.com/tests/chisquare/), accessed on 7 July 2026. A *p*-value < 0.05 was considered significant. For multiple pairwise comparisons, the Bonferroni correction was applied.

### 2.7. GenBank Accession Numbers

Nucleotide sequences obtained in the study are available in the GenBank database under accession numbers: PX117416-PX117422, PX117508, PX117514-PX117522, PZ322721-PZ322722, PZ322748-PZ322749 for *B. afzelii*; PX117423-PX117480, PX117509-PX117513, PX117523-PX117553, PZ322723-PZ322737, PZ322750-PZ322761 for *B. bavariensis*; PX117481-PX117507, PX117554-PX117564, PZ322738-PZ322747, PZ322762-PZ322770 for *B. garinii*; PX117565-PX117571, PZ363802-PZ363810 for *B. miyamotoi*; PX117572-PX117578, PZ343136-PZ343152, PZ363811-PZ363840 for *Borrelia* spp.

## 3. Results

### 3.1. Sampling

The study included 3107 Ixodidae ticks, which were identified to the species level using morphological criteria and genetic analysis. The ticks were collected from vegetation in the territory of two regions of the mainland part of the Russian Far East (Khabarovsk Territory and Amur province), on four islands of the Primorsky Territory, and on Sakhalin Island. The ticks belonged to three genera: *Ixodes* (n = 1450), *Haemaphysalis* (n = 1379), and *Dermacentor* (n = 278) (Table 2).

*Ixodes* spp. ticks were represented by two species, *I. persulcatus* (n = 1199) and *I. pavlovskyi* (n = 214), and their interspecific hybrids (n = 37). *Ixodes pavlovskyi* was found mainly on Russky Island (n = 211) and rarely on Putyatin Island (n = 3). The relative proportion of *I. persulcatus* and *I. pavlovskyi* varied depending on location. On Russky Island, *I. pavlovskyi* accounted for 40.0% of all *Ixodes* spp., ranging from 17.4% (site Rus1) to 77.6% (site Rus3) (Table S1). Notably, *I. persulcatus*/*I. pavlovskyi* interspecific hybrids were identified in all sites where parent species co-occurred, namely on Russky Island (n = 34) and Putyatin Island (n = 1). In addition, two ticks with hybrid genotypes were identified in Khabarovsk Territory, although *I. pavlovskyi* was not found among the examined ticks (Table S1).

Among hybrids, 27 individuals carried ITS2 hybrid fragments; these hybrid variants could have resulted from crossing *I. persulcatus* and *I. pavlovskyi* ticks and correspond to the genotypes of F1 offspring. The remaining ten hybrids showed mitochondrial and nuclear loci belonging to different species, suggesting repeated backcrossing with parental species and thus representing F2 progeny.

Among *Haemaphysalis* spp. ticks, *H. concinna* (n = 610) and *H. japonica* (n = 631) were found on different islands of the Primorsky Territory and in the mainland regions, whereas *H. longicornis* (n = 138) was found only on Askold Island, where it was predominant. Ticks of the genus *Dermacentor* in the Russian Far East were represented by a single species, *D. silvarum* (Table 2).

### 3.2. Detection and Genotyping of Borrelia burgdorferi Sensu Lato

Overall, *B. burgdorferi* s.l. was found in 432 (13.9%) of 3107 examined ticks. The prevalence of *B. burgdorferi* s.l. significantly varied among tick species; the prevalence reached 23.8–30.5% for *Ixodes* spp., versus only 0.7% and 0.3% for *H. concinna* and *H. japonica*, respectively (Table 2). *B. burgdorferi* s.l. was not found in any of 138 tested *H. longiconis* and 278 *D. silvarum*.

Three genospecies from the *B. burgdorferi* s.l. complex were identified: *B. afzelii*, *B. bavariensis*, and *B. garinii*. Their prevalence varied substantially between tick species. Thus, the total prevalence of *B. afzelii* and *B. bavariensis* in *I. persulcatus* (6.8% and 23.6%, respectively) was significantly higher (*p* < 0.0001 for both comparisons) than their prevalence in *I. pavlovskyi* (0.9% and 2.3%). In contrast, *B. garinii* was significantly more common (*p* < 0.0001) in *I. pavlovskyi* (20.6%) than in *I. persulcatus* (2.7%). The prevalence of all *B. burgdorferi* s.l. genospecies in hybrids was intermediate compared to both parental species (Table 2).

Russky Island was the only study site with a comparable proportion of *I. persulcatus* and *I. pavlovskyi*. On this island, the prevalence of different *B. burgdorferi* s.l. genospecies also differed significantly between two *Ixodes* species. *B. afzelii* occurred significantly more frequently (*p* = 0.0015) in *I. persulcatus* (6.7%; 19/283) than in *I. pavlovskyi* (0.9%; 2/211). Similarly, *B. bavariensis* was significantly more prevalent (*p* < 0.0001) in *I. persulcatus* (17.7%; 50/283) compared to *I. pavlovskyi* (2.4%; 5/211). In contrast, *B. garinii* was significantly more common (*p* < 0.0001) in *I. pavlovskyi* (20.9%; 44/211) than in *I. persulcatus* (2.8%; 8/283) (Table 2).

High abundance of *I. persulcatus* was observed in three regions (Khabarovsk Territory, Russky Island, and Sakhalin); in each of these regions, *I. persulcatus* significantly more frequently (*p* < 0.0001) harbored *B. bavariensis* (25.9%, 17.7%, and 30.8%, respectively) than *B. afzelii* (7.7%, 6.7%, and 3.7%) or *B. garinii* (2.8%, 2.8%, and 0%) (Table 2). Mixed infections were found in 26 *I. persulcatus* from Khabarovsk Territory and six *Ixodes* spp. from Russky Island. Of these, 31 ticks harbored both *B. afzelii* and *B. bavariensis*, and one tick harbored *B. afzelii* and *B. garinii*.

**Figure 2:**
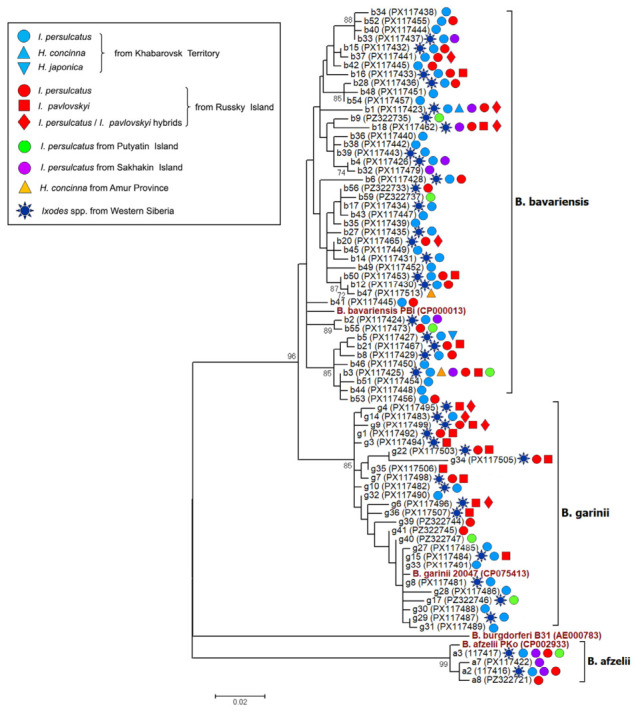
Phylogenetic tree generated using the ML method under the Tamura–Nei with Gamma distribution (TN93+G) substitution model, based on *p83/100* gene sequences (276–366 bp) of *Borrelia burgdorferi* s.l. identified in Ixodidae ticks. Source information for *B. burgdorferi* s.l. isolates is provided in the legend. The scale bar represents 0.02 nucleotide substitutions per site. Bootstrap values exceeding 70% are indicated on the nodes.

The identified *B. burgdorferi* s.l. specimens were genetically characterized by the *p83/100* and *clp*A genes. Based on the *p83/100* gene, four *B. afzelii*, 44 *B. bavariensis*, and 25 *B. garinii* haplotypes were found, of which two *B. afzelii***,** 23 *B. bavariensis*, and ten *B. garinii* haplotypes were novel. Most of the identified haplotypes (two *B. afzelii*, 22 *B. bavariensis*, and 23 *B. garinii*) were detected only in one of the Far Eastern regions. The phylogenetic analysis demonstrated that *B. afzelii* sequences formed a highly supported monophyletic clade, whereas all *B. garinii* sequences with a high level of support clustered within a common *B. bavariensis*/*B. garinii* clade (Figure 2).

**Figure 3:**
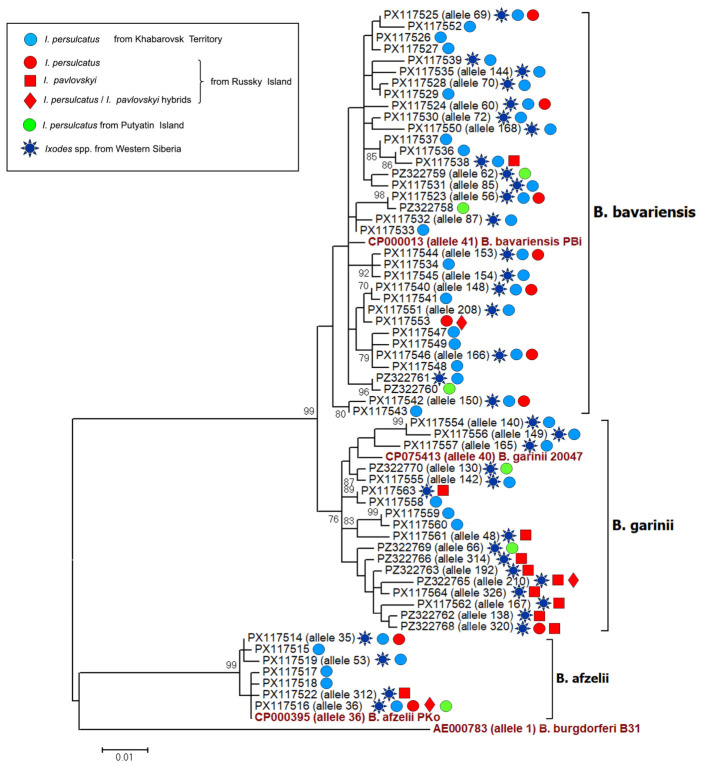
Phylogenetic tree generated using the ML method under the Tamura 3-parameter with Gamma distribution (T92+G) substitution model, based on *clp*A gene sequences (579 bp) of *Borrelia burgdorferi* s.l. identified in Ixodidae ticks. Source information for *B. burgdorferi* s.l. isolates is provided in the legend. The scale bar represents 0.01 nucleotide substitutions per site. Bootstrap values exceeding 70% are indicated on the nodes.

Based on the *clp*A gene, seven *B. afzelii*, 35 *B. bavariensis*, and 18 *B. garinii* haplotypes were found. Of these, three *B. afzelii*, 16 *B. bavariensis*, and three *B. garinii* haplotypes were novel, whereas the other haplotypes corresponded to known *clp*A alleles from the PubMLST website or to sequences from the GenBank database. Most *clp*A gene haplotypes (four *B. afzelii*, 27 *B. bavariensis*, and 18 *B. garinii*) were detected only in one of the Far Eastern regions. Based on the phylogenetic analysis, all obtained *B. afzelii*, *B. bavariensis*, and *B. garinii* sequences formed separate monophyletic clades. Reliable clustering based on geography within each clade was not observed (Figure 3).

### 3.3. Detection and Genotyping of Borrelia from RF Group

Borreliae from the RF group were found in different tick species (*I. persulcatus*, *I. pavlovskyi*, *H. concinna*, and *H. japonica*) from all studied regions. Based on *glp*Q gene analysis, the identified *Borrelia* spp. belonged to two genetically distinct clusters—*B. miyamotoi* and RF borreliae genetically close to the *Borrelia* sp. HM strain isolated from *H. megaspinosa* in Japan [10].

**Figure 4:**
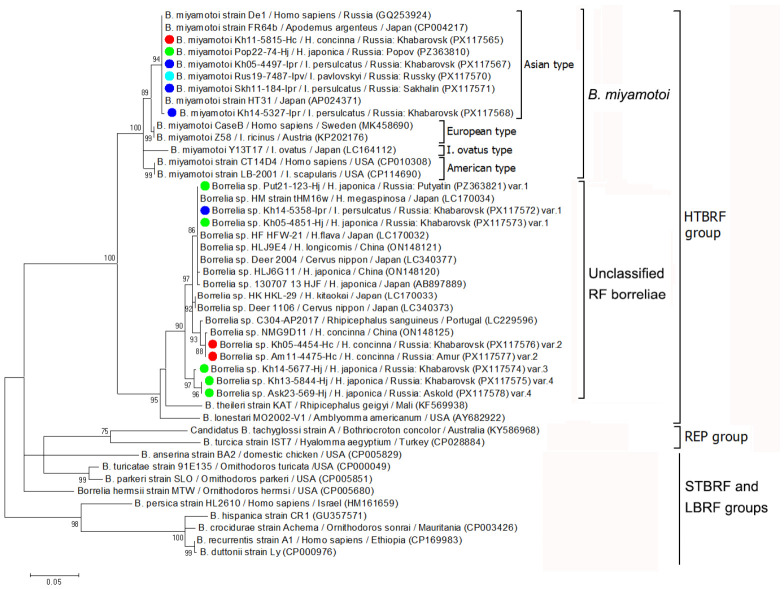
Phylogenetic tree generated using the ML method under the General Time Reversible with invariant sites (GTR+I) substitution model, based on *glp*Q gene sequences (359 bp) of RF borreliae identified in Ixodidae ticks. Samples from *I. persulcatus*, *I. pavlovskyi*, *H. concinna*, and *H. japonica* are marked with blue, light blue, red, and green circles, respectively. The scale bar represents 0.05 nucleotide substitutions per site. Bootstrap values exceeding 70% are indicated on the nodes.

*Borrelia miyamotoi* was found in 44 (1.4%) of the analyzed ticks, including 38 (3.2%) *I. persulcatus*, as well as single *I. pavlovskyi*, *H. concinna*, and *H. japonica* (Table 3). All *B. miyamotoi* specimens were genotyped by the *glp*Q gene; in addition, four specimens from *I. persulcatus* were characterized by the *fla*B gene. Most *glpQ* gene sequences were identical to Asian type *B. miyamotoi*, including the type strain HT31 (AP024371); one sequence from *I. persulcatus* in Khabarovsk Territory (PX117568) differed by a single nucleotide substitution (Figure 4). Similarly, all determined *fla*B gene sequences were identical and corresponded to *B. miyamotoi* sequences of the Asian type (AP024371).

The RF borreliae not belonging to validated species were identified in 28 (4.4%) *H. japonica*, four (0.7%) *H. concinna*, and one (0.1%) *I. persulcatus* (Table 3). Across tick species, RF borreliae were found significantly more frequently (*p* < 0.0001) in *H. japonica* than in other tick species. Due to the low prevalence of RF *Borrelia*, comparison between regions cannot be considered reliable.

Based on *glp*Q, *flab*, and 16S rRNA gene sequences, the RF *Borrelia* isolates were divided into four variants (variants 1–4); the obtained sequences of each tested gene differed between variants but were identical within each variant. Variant 1 was the most common, being found in 25 *H. japonica* and a single infected *I. persulcatus*. Variant 2 was found in all four infected *H. concinna*, while variants 3 and 4 were determined in one and two *H. japonica*, respectively (Table 3).

The obtained *glp*Q gene sequences differed by 4–12 nucleotide substitutions (96.7–98.9% identity) among different variants. Variants 1 and 2 were identical to or differed by a single substitution from the closest sequences from *H. flava*, *H. megaspinosa*, *H. longicornis*, *H. concinna*, and wild deer from Japan and China (LC170032, LC170034, ON148121, ON148125, and LC340377). Variants 3 and 4 differed from the closest sequence from *H. kitaokai* (LC170033) by 8 and 10 substitutions (97.8 and 97.2% identity), respectively (Figure 4).

**Figure 5:**
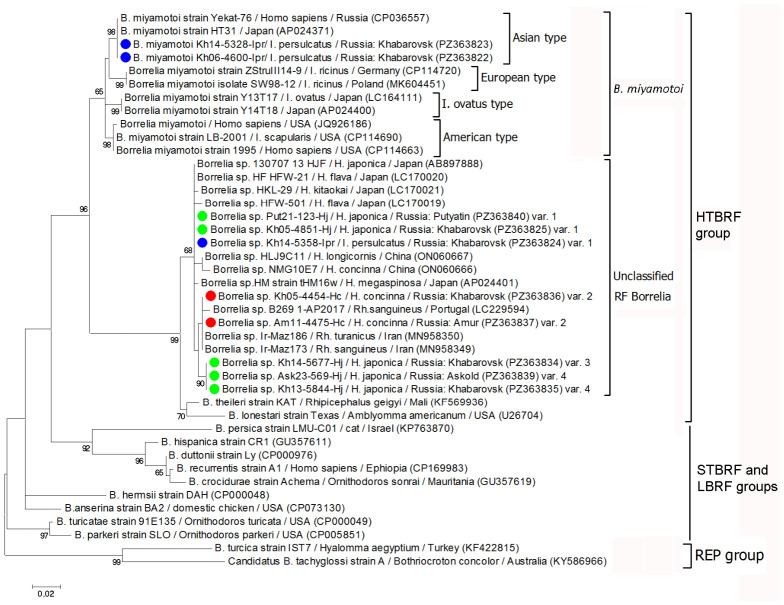
Phylogenetic tree generated using the ML method under the Tamura 3-parameter with Gamma distribution (T92+G) substitution model, based on *fla*B gene sequences (379–397 bp) of RF borreliae, identified in Ixodidae ticks. Samples from *I. persulcatus*, *H. concinna*, and *H. japonica* are marked with blue, red, and green circles, respectively. The scale bar represents 0.02 nucleotide substitutions per site. Bootstrap values exceeding 70% are indicated on the nodes.

**Figure 6:**
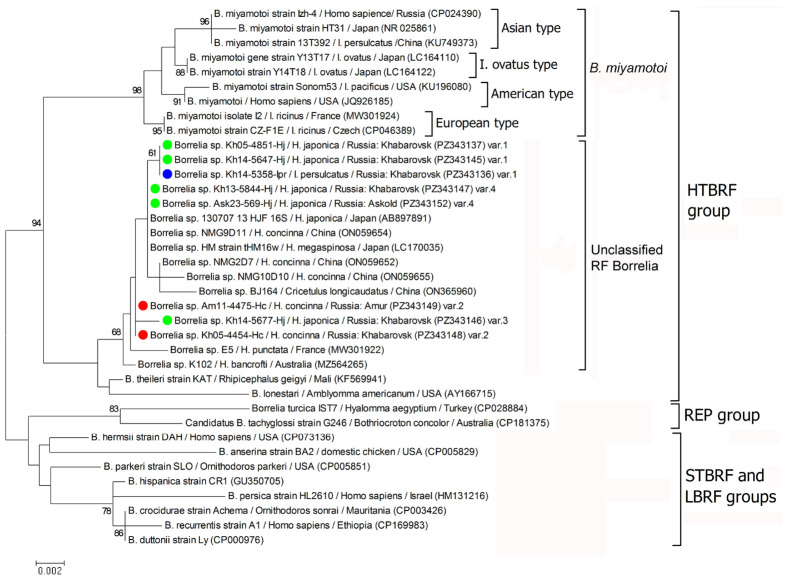
Phylogenetic tree generated using the ML method under the Hasegawa-Kishino-Yano with Gamma distribution (NKY+G) substitution model, based on 16S rRNA gene sequences (1221 bp) of RF borreliae, identified in Ixodidae ticks. Samples from *I. persulcatus*, *H. concinna*, and *H. japonica* are marked with blue, red, and green circles, respectively. The scale bar represents 0.002 nucleotide substitutions per site. Bootstrap values exceeding 60% are indicated on the nodes.

The *fla*B and 16S rRNA genes were less variable, differing among variants by 1–4 substitutions (98.9–99.7% identity for *flaB*; 99.7–99.9% identity for 16S rRNA). The *fla*B gene sequences of variant 1 matched those of isolates from *H. japonica* (AB897888) and *H. flava* (LC170020) from Japan. The *fla*B sequences of variant 2 were identical to those from *Rhipicephalus turanicus* (MN958350), *Rhipicephalus bursa* (MN958351), and *Rhipicephalus sanguineus* (MN958349) from Iran [18]. Similar to the *glp*Q gene, the *fla*B sequences from variants 3 and 4 were novel and differed from the closest sequences from *Rhipicephalus* spp. from Iran (MN958349-MN958351) by 3 and 4 nucleotide substitutions (99.2% and 99.0% identity), respectively (Figure 5). For the 16S rRNA gene, variant 4 matched the *Borrelia* sp. HF strain (AP024401); variants 1–3 differed from this sequence by 1, 1, and 3 substitutions, respectively (Figure 6).

The constructed phylogenetic trees based on all three examined genetic loci showed similar topology. In all trees, the HTBRF group is reliably subdivided into two large clades. One clade included *B. miyamotoi* isolates, subdivided into four well-supported groups corresponding to the *B. miyamotoi* type. The other clade contained two valid species (*B. theileri* and *B. lonestari*) and a well-supported monophyletic group of unclassified borreliae previously reported from *Haemaphysalis* spp. and *Rhipicephalus* spp. ticks in Japan, China, Iran, and Portugal. All obtained *B. miyamotoi* sequences from this study belong to the group formed by the Asian type of this agent, while the obtained sequences of new RF *Borrelia* belong to the large cluster of unclassified RF *Borrelia* (Figure 4, Figure 5 and Figure 6).

## 4. Discussion

This study provides the first comprehensive investigation of *Borrelia* spp. infection in Ixodidae ticks in the Russian Far East, including detection of spirochetes from both LB and RF groups in various tick species inhabiting the mainland and island locations, as well as detailed genetic analysis of identified borreliae. Previously, *B. burgdorferi* s.l. prevalence in Ixodidae ticks in the Russian Far East was examined in several studies [46,47]; however, reliable information on the species composition of borreliae in the studied region is absent.

The Ixodidae tick population in the Russian Far East includes a wide range of tick species belonging to three genera: *Ixodes*, *Haemaphysalis*, and *Dermacentor* [35,36,37,38,39,40]. *Ixodes* spp. ticks are the primary vectors of *B. burgdorferi* s.l. and *B. miyamotoi*, whereas *Haemaphysalis* spp. have been shown to harbor unclassified borreliae of the RF group [7,10,13].

In the studied region, the *Ixodes* spp. population is represented by *I. persulcatus*, widely distributed in Asian Russia, and *I. pavlovskyi*, which always co-exists with *I. persulcatus* and inhabits two remote disjunct areas—in Western Siberia and the Far East [30,36,41,42,43,44,48,49,50,51]. Notably, these sympatric areas differ principally from each other. First, *I. pavlovskyi* ticks from Western Siberia and the Far East belong to different subspecies—*I. pavlovskyi occidentalis* and *I. pavlovskyi pavlovskyi*, respectively. Second, in Western Siberia, the distribution range of *I. pavlovskyi* has expanded dramatically in recent decades from mountainous regions (Altai, Kuznetsk Alatau, and the Salair Ridge) into vast lowlands of Novosibirsk and Tomsk provinces; in some locations, *I. pavlovskyi* has almost completely displaced *I. persulcatus* [23,52,53]. In contrast, there is no reliable information about the expansion of the *I. pavlovskyi* distribution range in the Far East.

In Western Siberia, natural *I. persulcatus*/*I. pavlovskyi* interspecific hybrids were found in all locations where both species coexist, with prevalence reaching 7–40% [42,53]. In the Far East, the prevalence of interspecific hybrids was lower (2–9% across sites). Nevertheless, as in Western Siberia, hybrid individuals were found in all sympatric sites (Table S1). As in Western Siberia, the identified hybrids were genetically diverse and corresponded to the genotypes typical of F1 and F2 hybrid progenies. Thus, both *I. pavlovskyi* subspecies are capable of crossing with *I. persulcatus* and producing fertile offspring.

Unexpectedly, two hybrid ticks were found in Khabarovsk Territory, where *I. pavlovskyi* itself was not detected. Both hybrid individuals were found in the southern part of the region, adjacent to Primorsky Territory, where *I. pavlovskyi* is consistently present [49]. This suggests that the south of Khabarovsk Territory is also most likely a sympatric area for *I. persulcatus* and *I. pavlovskyi*.

Different *Ixodes* ticks are known to be associated with specific *B. burgdorferi* s.l. genospecies [7]. The results of this study are consistent with those previously conducted in the Urals and Western Siberia [23,32,33]. In all studied regions of Asian Russia (Urals, Western Siberia, and the Far East), *I. persulcatus* was significantly more frequently infected with *B. afzelii* and *B. bavariensis* and less often with *B. garinii* compared to *I. pavlovskyi*. Notably, *B. garinii* was not found in regions where *I. pavlovskyi* or hybrid ticks were absent—Sakhalin and northern Khabarovsk Territory in the Far East (Table S1) and Omsk province of Western Siberia [54].

MLST is a robust and widely adopted method for *B. burgdorferi* s.l. genotyping, which has contributed to the discovery of several new species, including *B. bavariensis* [55,56]. However, its application to field-collected ticks is often limited by the low DNA yield from individual specimens. Therefore, genotyping of *Borrelia* in natural tick populations typically relies on the analysis of several (or even just one) genes encoding housekeeping or outer membrane proteins [33,54,57,58]. In this study, we used the outer membrane protein (*p83/100*) and housekeeping (*clp*A) genes for genetic characterization of *B. burgdorferi* s.l. from the Russian Far East. The reliability of these markers for species identification has been validated using a reference panel of Asian *B. burgdorferi* s.l. strains [43] and confirmed in our subsequent studies [23,24,42].

Consistent with this approach, we detected high haplotype diversity at both the *p83/100* and *clpA* loci among Far Eastern *B. burgdorferi* s.l. isolates, especially for *B. bavariensis* and *B. garinii* (Figure 2 and Figure 3). A substantial proportion of haplotypes identified in this study were novel and, to date, have been found only in the Far Eastern region. Most of the remaining haplotypes were found in Western Siberia; however, some were found in Asian countries and Europe. Notably, several *B. afzelii* and *B. bavariensis* haplotypes (e.g., a2, a3, b1, and b3 by the p83/100 gene) were widely distributed, being shared across 3–5 of the studied Far Eastern regions as well as in other regions of Russia (Figure 2).

Comparison of *p83/100* gene sequences of *B. burgdorferi* s.l. from this and previous studies [23,24,42,54] demonstrated that two *B. afzelii*, 20 *B. bavariensis*, and 13 *B. garinii* haplotypes were found in both Western Siberia and the Far East. Similarly, based on the *clp*A gene, four *B. afzelii*, 13 *B. bavariensis*, and nine *B. garinii* haplotypes were found in both these regions (Figure 2 and Figure 3). The obtained results indicate that the transfer of *B. burgdorferi* s.l. strains between the Far East and Western Siberia occurs frequently. This is unsurprising for *B. afzelii* and *B. bavariensis*, whose main carrier, *I. persulcatus*, has a continuous range from Siberia to the Far East. The main reservoir hosts of these agents are small mammals; therefore, *B. afzelii* and *B. bavariensis* strains can easily spread over long distances via both reservoir hosts and infected *I. persulcatus* ticks attached to their hosts.

For *B. garinii* strains, the dispersal route is less obvious because of a large distance between West Siberian and Far Eastern distribution areas of *I. pavlovskyi*, which is thought to play a major role in the transmission of *B. garinii* in Asian Russia. The reservoir hosts of *B. garinii* are birds, suggesting that long-distance dispersal likely occurs via migratory birds. However, some *B. garinii* strains may be adapted to *I. persulcatus* and, like *B. afzelii* and *B. bavariensis*, spread over long distances via infected *I. persulcatus* and short-distance migrating birds. This possibility is supported by the finding that several *B. garinii* variants (*clp*A alleles 140, 142, 149, and 165) were found only in *I. persulcatus* from different regions: Khabarovsk Territory and Urals of Russia [33] and Japan (Figure 3). Unfortunately, the lack of genetic studies of *B. burgdorferi* s.l. in Eastern Siberia (between the *I. pavlovskyi* ranges) prevents a reliable test of this hypothesis.

Among borreliae of the RF group, only *B. miyamotoi* is reliably associated with *Ixodes* spp. ticks [13]. As expected, we detected *B. miyamotoi* belonging to the Asian type in *I. persulcatus* and *I. pavlovskyi* with a low prevalence (0.9–4.4% for different regions) (Table 3), which corresponds to the previous reports from Asian Russia [23,33,34,46,59].

Both LB borreliae (*B. afzelii* and *B. bavariensis*) and *B. miyamotoi* were found only occasionally in *Haemaphysalis* spp., with prevalence below 1% (Table 2 and Table 3), which is consistent with previous data [46,60,61]. Nevertheless, there is no reliable evidence that *Haemaphysalis* spp., or any tick genus other than *Ixodes*, serve as competent vectors for LB spirochetes or *B. miyamotoi* [7,13]. The sporadic presence of these pathogens in *Haemaphysalis* spp. most likely reflects accidental spillover of pathogens between tick genera, rather than indicating vector competence.

The most remarkable discovery of this study is the finding of a diverse group of RF borreliae, not assignable to any validated species, in *H. japonica* (4.4%) and less frequently in *H. concinna* (0.7%) and *I. persulcatus* (0.1%). Based on *glp*Q, *fla*B, and 16S rRNA sequences, these isolates formed four distinct variants. Although sequence analysis suggested an association of isolates of variant 2 with *H. concinna*, their comparison with isolates from other regions did not support this association. Indeed, the obtained sequences of the *fla*B gene of variant 2 were identical to those from *Rhipicephalus* spp. from Iran [18] but differed by two substitutions (99.5% identity) from *H. concinna* isolates from China (ON060066) [62]. However, since isolates from different studies were often genotyped at different genetic loci, detailed comparison of unclassified RF borreliae with respect to tick species is hindered.

With the exception of one isolate detected in an *I. persulcatus* in this study, unclassified RF borreliae have been found only in *Haemaphysalis* and *Rhipicephalus* ticks [10,17,18,62]. This group forms a well-supported monophyletic cluster in all constructed phylogenetic trees, which in turn forms a common clade with *B. theileri* and *B. lonestari* from *Amblyomma* and *Rhipicephalus* ticks. Thus, the HTBRF group of RF borreliae is reliably divided into two major clades, associated with different tick genera. One clade comprises *B. miyamotoi* isolates, i.e., RF borreliae associated with *Ixodes* spp., while the other clade includes RF borreliae associated with *Haemaphysalis* spp., *Rhipicephalus* spp., and *Amblyomma* spp., i.e., with metastriate ticks (Figure 4, Figure 5 and Figure 6). We propose that the group of unclassified RF borreliae is likely to be formally described as new species in the future, especially given that one isolate from this group has been successfully cultured [10]. Pathogenic properties of RF borreliae from this group are unknown.

Our study has several limitations. First, sampling periods and the sample sizes varied across regions, which may affect the comparability of prevalence data. Second, genotyping of *B. burgdorferi* s.l. was not performed at both loci for every positive sample, potentially limiting the resolution of haplotype comparisons. Finally, the absence of MLST data for the newly identified borreliae from the RF group means that their exact phylogenetic position remains to be firmly established. Nevertheless, the combined use of *glp*Q, *fla*B, and 16S rRNA gene sequences provided a reliable basis for their preliminary classification.

## 5. Conclusions

This study reports for the first time the high genetic diversity of borreliae from the LB and RF groups in the Russian Far East. Overall, *B. afzelii*, *B. bavariensis*, and *B. garinii* from the LB group, pathogenic *B. miyamotoi* from the RF group, and a group of genetically diverse unclassified RF borreliae were identified. Our findings confirm a strong association of *B. burgdorferi* s.l. and *B. miyamotoi* with *Ixodes* spp., while unclassified RF borreliae were predominantly associated with *Haemaphysalis* spp. Within the LB group, *B. afzelii* and *B. bavariensis* were significantly more common in *I. persulcatus*, whereas *B. garinii* prevailed in *I. pavlovskyi*, a pattern consistent with previous observations in Western Siberia. All *B. miyamotoi* isolates were highly conserved and belonged to the Asian type. In contrast, the unclassified RF borreliae exhibited notable genetic variability and clustered with RF borreliae from *Haemaphysalis* and *Rhipicephalus* ticks. Notably, this is the first report of unclassified RF borreliae in Russia, expanding the known geographic range of this group.

## Figures and Tables

**Table 2:** Results of PCR detection and species determination of *Borrelia burgdorferi* s.l. in ticks.

Region/Year	Tick Species	No of Tested Ticks	No (%) of Ticks Containing DNA of				No (%) of Ticks with Bbsl Mixed Infection
			Total Bbsl	B.a. *	B.bav. *	B.gar. *	
Khabarovsk	*I. persulcatus*	753	248 (32.9)	58 (7.7)	195 (25.9)	21 (2.8)	26 (3.5)
Territory/2005–2017	Hybrids	2	0	0	0	0	0
	*H. concinna*	241	2 (0.8)	1 (0.4)	1 (0.4)	0	0
	*H. japonica*	446	2 (0.4)	0	2 (0.4)	0	0
	*D. silvarum*	203	0	0	0	0	0
Amur province/2011	*H. concinna*	198	2 (1.0)	0	2 (1.0)	0	0
	*D. silvarum*	75	0	0	0	0	0
Russky Island/	*I. persulcatus*	283	72 (25.4)	19 (6.7)	50 (17.7)	8 (2.8)	5 (1.8)
2019–2022	*I. pavlovskyi*	211	51 (24.1)	2 (0.9)	5 (2.4)	44 (20.9)	0
	Hybrids	34	9 (26.5)	1 (2.9)	4 (11.8)	5 (14.7)	1 (2.9)
	*H. concinna*	36	0	0	0	0	0
	*H. japonica*	52	0	0	0	0	0
Putyatin Island/2021	*I. persulcatus*	56	9 (16.1)	1 (1.8)	5 (8.9)	3 (5.4)	0
	*I. pavlovskyi*	3	0	0	0	0	0
	Hybrids	1	0	0	0	0	0
	*H. concinna*	117	0	0	0	0	0
	*H. japonica*	15	0	0	0	0	0
Askold Island/2023	*H. japonica*	94	0	0	0	0	0
	*H. longicornis*	138	0	0	0	0	0
Popov Island/2022	*H. concinna*	18	0	0	0	0	0
	*H. japonica*	24	0	0	0	0	0
Sakhalin Island/2011	*I. persulcatus*	107	37 (34.6)	4 (3.7)	33 (30.8)	0	0
All regions	*I. persulcatus*	1199	366 (30.5)	82 (6.8)	283 (23.6)	32 (2.7)	31 (2.6)
	*I. pavlovskyi*	214	51 (23.8)	2 (0.9)	5 (2.3)	44 (20.6)	0
	Hybrids	37	9 (24.3)	1 (2.7)	4 (10.8)	5 (13.5)	1 (2.6)
	*H. concinna*	610	4 (0.7)	1 (0.2)	3 (0.5)	0	0
	*H. japonica*	631	2 (0.3)	0	2 (0.3)	0	0
	*H. longicornis*	138	0	0	0	0	0
	*D. silvarum*	278	0	0	0	0	0
	**All species**	**3107**	**432 (13.9)**	**86 (2.8)**	**297 (9.6)**	**81 (2.6)**	**32 (1.0)**

* Including cases of mixed infections. Abbr. Bbsl—*B. burgdorferi* s.l., B.a.—*B. afzelii*, B.bav.—*B. bavariensis*, B.gar.—*B. garinii*; Hybrids—*I. persulcatus*/*I.pavlovskyi* interspecific hybrids.

**Table 3:** Results of PCR detection and genotyping of RF borreliae in ticks.

Region/Year	Tick Species	No of Tested Ticks	No (%) of Ticks Containing DNA of					
			B.m.	New RF *Borrelia*				
				All Variants	var. 1	var. 2	var. 3	var. 4
Khabarovsk	*I. persulcatus*	753	33 (4.4)	1 (0.1)	1	0	0	0
Territory/2005–2017	Hybrids	2	0	0	-	-	-	-
	*H. concinna*	241	2 (0.8)	1 (0.4)	0	1	0	0
	*H. japonica*	446	1 (0.2)	26 (5.8)	24	-	1	1
	*D. silvarum*	203	0	0	-	-	-	-
Amur province/2011	*H. concinna*	198	0	3 (1.5)	0	3	0	0
	*D. silvarum*	75	0	0	-	-	-	-
Russky Island/	*I. persulcatus*	283	4 (1.4)	0	-	-	-	-
2019–2022	*I. pavlovskyi*	211	2 (0.9)	0	-	-	-	-
	Hybrids	34	0	0	-	-	-	-
	*H. concinna*	36	0	0	-	-	-	-
	*H. japonica*	52	0	0	-	-	-	-
Putyatin Island/2021	*I. persulcatus*	56	0	0	-	-	-	-
	*I. pavlovskyi*	3	0	0	-	-	-	-
	Hybrids	1	0	0	-	-	-	-
	*H. concinna*	117	0	0	-	-	-	-
	*H. japonica*	15	0	1 (6.7)	1	0	0	0
Askold Island /2023	*H. japonica*	94	0	1 (1.1)	0	0	0	1
	*H. longicornis*	138	0	0	-	-	-	-
Popov Island/2022	*H. concinna*	18	0	0	-	-	-	-
	*H. japonica*	24	1 (4.2)	0	-	-	-	-
Sakhalin Island/2011	*I. persulcatus*	107	1 (0.9)	0	-	-	-	-
All regions	*I. persulcatus*	1199	38 (3.2)	1 (0.1)	1	0	0	0
	*I. pavlovskyi*	214	2 (0.9)	0	-	-	-	-
	Hybrids	37	0	0	-	-	-	-
	*H. concinna*	610	2 (0.3)	4 (0.7)	0	4	0	0
	*H. japonica*	631	2 (0.3)	28 (4.4)	25	0	1	2
	*H. longicornis*	138	0	0	-	-	-	-
	*D. silvarum*	278	0	0	-	-	-	-
	**All species**	**3107**	**44 (1.4)**	**33 (1.1)**	**26**	**4**	**1**	**2**

Abbr. B.m.—*B. miyamotoi*, Hybrids—*I. persulcatus*/*I.pavlovskyi* interspecific hybrids.

## Data Availability

The original contributions presented in this study are included in the article and Supplementary Material. Further inquiries can be directed to the corresponding author.

## Supplementary Materials

The following supporting information can be downloaded at: https://www.mdpi.com/article/10.3390/pathogens15070775/s1, Table S1: Results of PCR detection and genotyping of *Borrelia burgdorferi* s.l. and RF *Borrelia* in ticks from different sampling sites.
